# Suppression of the epithelial-mesenchymal transition by SHARP1 is linked to the NOTCH1 signaling pathway in metastasis of endometrial cancer

**DOI:** 10.1186/1471-2407-14-487

**Published:** 2014-07-05

**Authors:** Yun Liao, Xiaoying He, Haifeng Qiu, Qi Che, Fangyuan Wang, Wen Lu, Zheng Chen, Meiting Qiu, Jingyun Wang, Huihui Wang, Xiaoping Wan

**Affiliations:** 1 Department of Obstetrics and Gynecology, International Peace Maternity & Child Health Hospital Affiliated to Shanghai Jiao Tong University School of Medicine, Shanghai, China; 2 Department of Obstetrics and Gynecology, Shanghai First People’s Hospital Affiliated to Shanghai Jiao Tong University School of Medicine, Shanghai, China; 3 Department of Obstetrics and Gynecology, Shanghai First Maternity and Infant Hospital Affiliated to Tong Ji University, No. 536, Changle Road, Shanghai 200080, China

**Keywords:** Endometrial cancer, Metastasis, SHARP1, EMT, NOTCH1

## Abstract

**Background:**

Mechanisms governing the metastasis of endometrial cancer (EC) are poorly defined. Recent data support a role for Enhancer-of-split and hairy-related protein 1 (SHARP1), a basic helix-loop-helix transcription repressor, in regulating invasiveness and angiogenesis of several human cancers. However, the role of SHARP1 in metastasis of EC remains unclear.

**Methods:**

Human EC cell lines (Ishikawa and HEC-1B) were used. SHARP1 was upregulated by lentivirus transduction, while intracellular domain of NOTCH1 (ICN) were upregulated by transient transfection with plasmids. Effects of SHARP1 on cell migration and invasion were evaluated by wound healing assay and transwell invasion assay. Experimental metastasis assay were performed in nude mice. Effects of SHAPR1 on protein levels of target genes were detected by western blotting. Furthermore, the association between SHARP1 and the NOTCH1/EMT pathway was further verified in EC tissue specimens by immunohistochemical analysis.

**Results:**

Overexpression of SHARP1 in EC cells inhibited cell migration, invasion, and metastasis. Exogenous SHARP1 overexpression affected the proteins levels of genes involved in EMT process and NOTCH1 signaling pathway. Upregulation of ICN in SHARP1-overexpressing Ishikawa cells induced cell migration and an EMT phenotype. Additionally, immunohistochemical analysis demonstrated that SHARP1 protein levels were lower in metastatic EC than in primary tumors, and statistical analysis revealed correlations between levels of SHARP1 and markers of EMT and NOTCH1 signaling pathway in human EC tissue specimen.

**Conclusions:**

This work supports a role for SHARP1 in suppressing EMT and metastasis in EC by attenuating NOTCH1 signaling. Therefore, SHARP1 may be a novel marker for lymphatic metastasis in EC patients.

## Background

Endometrial cancer (EC) is the most common gynecological malignancy worldwide. In the United States, it ranks fourth among female malignancies, with an estimated 49,560 new cases and 8190 deaths in 2013 [1]. Despite advances in surgical treatment for early-stage EC (with or without adjuvant therapy), treatment of advanced EC is less effective and prognosis is poor [2,3]. The primary reasons for this poor prognosis are metastasis and recurrence, with a median survival of only 7–12 months [4]. It is therefore important to characterize the molecular mechanisms underlying EC metastasis.

Enhancer-of-split and hairy-related protein 1 (SHARP1), which is also called basic helix-loop-helix family, member e41 (BHLHE41) or differentially expressed in chondrocytes 2 (DEC2), is a member of the transcriptional repressor subfamily of basic helix-loop-helix transcription factors [5,6] and is expressed in various embryonic and adult tissues [7,8]. Emerging evidence suggests that SHARP1 is involved in tumor progression [9-11]. Low-level expression of SHARP1 is associated with the tumor stage in EC [12], and SHARP1 suppresses breast cancer metastasis by degrading hypoxia-inducible factor 1α (HIF-1α) [11]. In our previous study, we also found that SHARP1 suppresses angiogenesis of endometrial cancer [13]. However, it remains unknown whether SHARP1 contributes to EC metastasis.

Increased cell invasion and migration are defining characteristics of metastatic cancer cells. Recent studies have shown that metastasis can be viewed as a reactivation of at least some aspects of the epithelial-mesenchymal transition (EMT), which normally is an embryonic process [14]. During EMT, epithelial cells undergo extensive alterations in gene expression, resulting in the loss of apical/basolateral polarity, the severing of intercellular adhesive junctions, and the degradation of basement-membrane components. In this way they become individual, non-polarized, motile, and invasive mesenchymal cells [15]. EMT is a dynamic process and is triggered by interactions between extracellular components (such as collagen) and secreted soluble factors (such as the wingless-type MMTV integration site family members (WNTs), transforming growth factor, beta 1, fibroblast growth factors, and epidermal growth factor [14]. Among these signaling pathways, the Notch/Snail/E-cadherin signaling pathway plays a critical role in inducing EMT [16]. High levels of NOTCH1 and its ligand jagged 1 are associated with poor prognosis in breast cancer, bladder cancer, leukemia, prostate cancer [17-19], and EC [20]. In addition, NOTCH1 induces an EMT phenotype and cell migration in pancreatic cancer [21,22] and intrahepatic cholangiocarcinoma [23]. These data prompted us to investigate the mechanisms by which the Notch/EMT signaling pathway is regulated in EC.

Here, we report that SHARP1 inhibits cell migration, invasion, and metastasis in EC cell lines, thereby reverting the EMT cellular phenotype. The effects of SHARP1 on EC involved the NOTCH1 signaling pathway. Re-activation of NOTCH1 signaling in SHARP1 overexpressing EC cells resulted in an EMT phenotype and induced cell migration. Furthermore, the association between SHARP1 and the NOTCH1/EMT pathway was further verified in EC tissue specimens. This work sheds light on the mechanisms and pathways by which EC becomes invasive and metastatic and identifies a potential therapeutic target for treating EC.

## Methods

### Ethics statement

This study was approved by the Human Investigation Ethics Committee of the International Peace Maternity and Child Hospital, which is affiliated with the Shanghai Jiao Tong University School of Medicine. EC specimens were collected after receiving written informed consent from patients. Animal research was carried out in strict accordance with Guideline for the Care and Use of Laboratory Animals of China. The protocol was approved by the Committee on the Ethics of Animal Experiments of the Obstetrical and Gynecological Hospital affiliated Fu Dan University (Permit Number: SYXK (hu) 2008–0064). All efforts were taken to minimize animal suffering.

### Patients and samples

Paraffin-embedded tissue samples were obtained from 15 patients with EC at the International Peace Maternity and Child Health Hospital during 2012 and 2013. The stages (I–IV) and histological grades (G1–G3) of these tumors were established according to criteria of the International Federation of Gynecology and Obstetrics surgical staging system (2009) [24]. None of the patients had undergone hormone therapy, radiotherapy, or chemotherapy before surgery.

### Immunohistochemistry (IHC) and assessments

Tissue sections (4 μm) from paraffin-embedded tissue specimens were dewaxed with xylene and then rehydrated using a graded alcohol series. Specimens were then incubated in 0.01 M sodium citrate (pH 6.0) for 20 min (for antigen retrieval), 3% hydrogen peroxide for 10 min (to block endogenous peroxidase activity), and 10% normal goat serum for 30 min (to block nonspecific staining). Sections were then incubated in a humidified chamber at 4°C overnight with primary antibodies against the following proteins, from Cell Signaling Technology (Beverly, MA): E-cadherin (1:400), N-cadherin (1:200), vimentin (1:100), and Snail (1:100); from Novus (Littleton, CO): SHARP1 (1:100); and from Abcam (Cambridge, UK): jagged 1 (1:500). Sections incubated with phosphate-buffered solution (PBS) only were used as negative control. Sections were then incubated with biotinylated secondary antibodies for 30 min, followed by streptavidin peroxidase for 15 min. Peroxidase activity was detected by applying 3,3’-diaminobenzidine tetrachloride. Each incubation step was performed at 37°C and was followed by three 5-min washes with PBS. Finally, sections were dehydrated in alcohol and cleared in xylene.

The intensity of IHC staining was scored independently by two pathologists who were blinded to the clinical and pathological data. They used a semiquantitative immunoreactivity score according to Remmele and Stegner [25], which takes into account both the intensity of the color reaction and the percentage of positive cells. The number of positive cells was graded as follows: 0 (< 5%), 1 (5–25%), 2 (26–50%), 3 (51–75%), and 4 (> 75%). Staining intensity was graded as follows: 0 (negative), 1 (weak), 2 (moderate), and 3 (strong). A final score was calculated by multiplying these two scores, generating an immunoreactivity score of 0–12.

### Cell culture and lentivirus transduction

Human EC cell lines (Ishikawa and HEC-1B) were obtained from the American Type Culture Collection (Manassas, VA). Ishikawa and HEC-1B cells were grown in Dulbecco’s modified Eagle medium (DMEM)/F12 (Gibco, Auckland, New Zealand) supplemented with 10% fetal bovine serum (Gibco, Carlsbad, CA). Cells were cultured in a 5% CO_2_ humidified incubator at 37°C. For stable expression of human SHARP1 in EC cells, SHARP1 coding sequences were cloned into lentiviral vectors with Ubi-MCS-3FLAG-SV40-EGFP using Gateway technology (Invitrogen, Carlsbad, CA) by GeneChem Biotech (Shanghai, China).

### Transient transfection

The expression plasmid encoding the intracellular domain of NOTCH1 (ICN), pIRES2-EGFP-ICN, and the empty plasmid, pIRES2-EGFP-NEG, were purchased from R&S Biotechnology (Shanghai, China). Transient transfection was performed using 70% confluent Ishikawa cells and Lipofectamine 2000 reagents (Invitrogen).

### RNA isolation and quantitative real-time PCR (qPCR)

Total RNA was extracted from cultured cells using Trizol (Invitrogen). First-strand cDNA was reverse-transcribed from 1 μg of total RNA using the Prime Script RT reagent kit (TaKaRa, Dalian, China). The resulting cDNA was analyzed by qPCR using SYBR Premix Ex Taq (TaKaRa). For all qPCR experiments, values on the *y* axis represent 2^(−ΔCt)^, where ΔCt is the difference between the gene-of-interest Ct and the β-actin Ct [26]. Primer sets are shown in Table 1. Data were obtained in triplicate from three independent experiments.

**Table 1:** qPCR primer sequences

**Gene**	**Sequence**
*SHARP1*	Forward 5′-GCATGAAACGAGACGACACC-3′
	Reverse 5′-CGCTCCCCATTCTGTAAAGC-3′
*β-actin*	Forward 5′-CATGTACGTTGCTATCCAGGC-3′
	Reverse 5′-CTCCTTAATGTCACGCACGAT-3′

### Western blotting

Cells were lysed in RIPA lysis buffer (Beyotime, Nanjing, China) with the protease inhibitor phenylmethanesulfonyl fluoride (Beyotime). Protein concentrations were determined using a BCA Protein Assay kit (Beyotime). Equal amounts of protein were loaded into each lane of an SDS-PAGE gel. Proteins were then separated with electrophoresis and transferred to a polyvinylidene fluoride membrane (Millipore, Billerica, MA). Each membrane was blocked with 5% skimmed milk for 2 h and then incubated with antibodies against SHARP1 (1:500), E-cadherin (1:1000), N-cadherin (1:1000), vimentin (1:1000), Snail (1:1000), NOTCH1 (1:2500; Epitomics, Burlingame, CA), jagged 1 (1:10000), HES1 (1:2000; Epitomics), or β-actin (1:5000; Epitomics) at 4°C overnight. Peroxidase-linked secondary antibodies against rabbit (1:5000; Epitomics) were used to detect bound primary antibodies. Probed proteins were detected by enhanced chemiluminescent reagents. The data have been normalized to β-actin expression by densitometry and statistical data from at least 3 experiments is graphed to provide additional validation of results.

### Wound-healing assays

Cells were grown to confluency as a monolayer and wounded by dragging a 10-μL pipette tip through the monolayer. Cells were washed to remove cellular debris and allowed to migrate for 12 h. Representative images were captured at 100× magnification. All experiments were repeated at least three times.

### Trans-well invasion assays

For trans-well invasion assays, the upper side of an 8-μm pore, 6.5-mm polycarbonate trans-well filter (Corning, New York, NY) chamber was uniformly coated with Matrigel basement membrane matrix (BD Biosciences, Bedford, MA) for 2 h at 37°C before cells were added. A total of 5 × 10^4^ cells were seeded into the top chamber of a trans-well filter (in triplicate) and incubated for 48 h. Invasive cells, which were on the lower side of the filter, were fixed in 4% paraformaldehyde, stained in 0.5% crystal violet (Beyotime), and counted using a microscope. A total of five fields were counted for each trans-well filter. Each field was counted and photographed at 200× magnification.

### In vivo experiments

Twelve 6-week-old female BALB/c mice were obtained from Shanghai Life Science Institute (Slac Laboratory Animal Co., Ltd., China) and randomly divided into two groups. Each mouse was injected intravenously through the tail vein with 1 × 10^6^ Ishikawa^Control^ or Ishikawa^SHARP1^ cells. Six weeks after the injection, mice were sacrificed and examined.

### Statistical analyses

All statistical analyses were performed using SPSS software, version 17.0 (Chicago, IL). Values represent the mean ± SD from one representative experiment of three independent experiments, each performed in triplicate. Data was analyzed using the unpaired Student’s *t*-test. The spearman’s correlation coefficient test was used for correlation detection. A *P*-value of < 0.05 was considered statistically significant. All experiments were performed at least three times.

## Results

### SHARP1 inhibits EC-cell migration and invasion in vitro and metastatic potential in vivo

**Figure 1:**
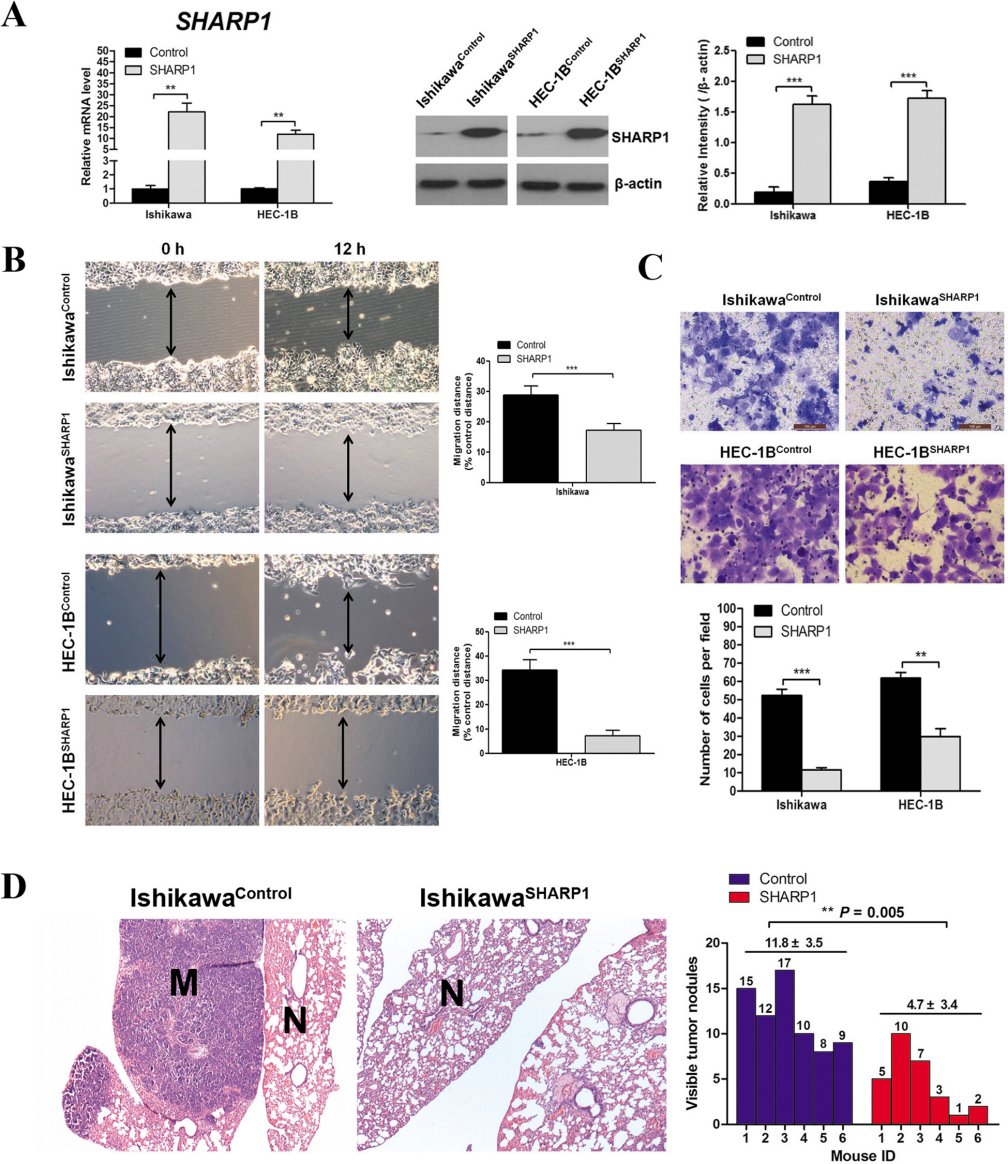
**SHARP1 inhibits migration, invasion, and metastasis of EC cells. (A)** Overexpression of SHARP1 in Ishikawa and HEC-1B cells revealed by qPCR (left) and western blotting (middle), and blots were further quantified by densitometry of triplicate blots (right), β-actin was included as an internal control. **(B)** Wound-healing assays for Ishikawa and HEC-1B cells. Representative images were obtained at 100× magnification. Graphs show the relative migration distance after 12 h incubation. **(C)** Trans-well invasion assays for Ishikawa and HEC-1B cells. Representative images were obtained at 200× magnification. Graph shows the number of invasive cells for each treatment group (averaged across five random images). **(D)** Ishikawa^Control^ or Ishikawa^SHARP1^ cells were injected intravenously into SCID mice. Sections of lung tissue were stained with hematoxylin and eosin. (M) metastatic tumor, (N) normal lung tissue. Images were obtained at 40× (left) magnification. Graph shows the number of visible tumor nodules within analyzed lung tissue (*n* = 6 per group). Data represent the mean ± SD from one representative experiment of three independent experiments. **P < 0.01, ***P < 0.001.

Migration and invasion are important prerequisites for tumor progression and metastasis. To determine the role of SHARP1 in EC progression, we stably transfected EC cell lines with a lentiviral vector expressing human SHARP1. Cells transfected with an empty vector served as the control. These cells lines were named Ishikawa^SHARP1^ or Ishikawa^Control^, and HEC-1B^SHARP1^ or HEC-1B^Control^. Efficient transfection was confirmed before cellular assays were performed (Figure 1A). Wound-healing and trans-well invasion assays both demonstrated that the migration and invasion capabilities of Ishikawa and HEC-1B cells were significantly suppressed by SHARP1 overexpression (Figure 1B and C).

To investigate the in vivo effect of SHARP1 on metastasis, we injected Ishikawa cells into severe combined immunodeficiency (SCID) mice. Mice injected with Ishikawa^SHARP1^ cells had fewer nodes per lung than mice injected with Ishikawa^Control^ cells (4.7 ± 3.4 versus 11.8 ± 3.5, *P* = 0.005). Histological studies confirmed that these lesions were caused by the extravasation and subsequent growth of Ishikawa cells in the lungs (Figure 1D). However, no metastasis in other organs was observed (data not shown). Our data indicated that SHARP1 is involved in controlling EC metastasis in vivo.

### SHARP1 overexpression reverses the EMT phenotype in EC cells

**Figure 2:**
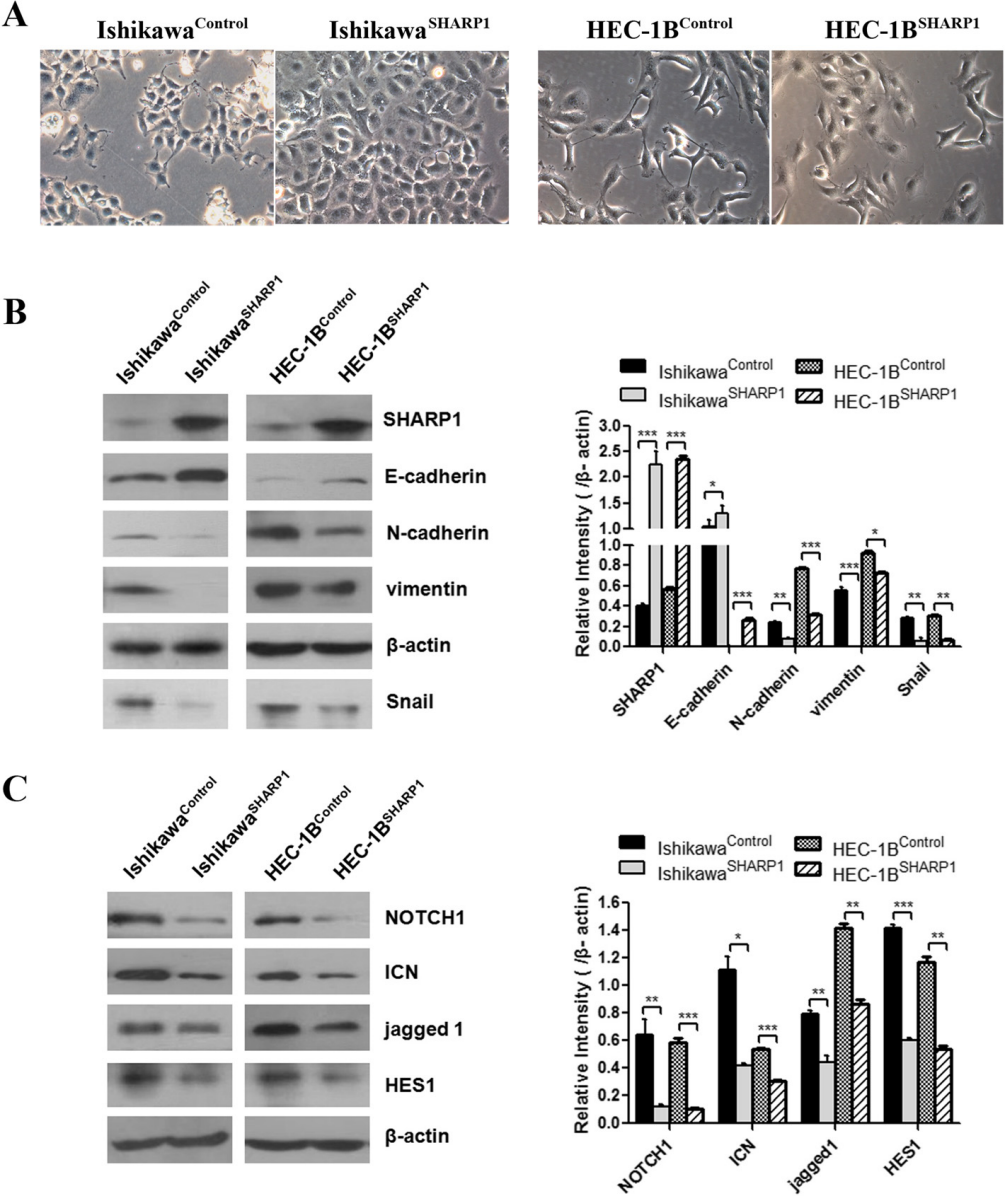
**SHARP1 overexpression reverses the EMT phenotype in EC cells. (A)** SHARP1 overexpression induced an epithelial morphology in Ishikawa and HEC-1B cells (magnification: 200×). **(B)** Western blot analysis of SHARP1 and EMT-related markers in Ishikawa and HEC-1B cells (left), and blots were further quantified by densitometry of triplicate blots (right). β-actin was included as an internal control. **(C)** Western blot analysis of the effects of SHARP1 overexpression on NOTCH1 signaling components in Ishikawa and HEC-1B cells (left), and blots were further quantified by densitometry of triplicate blots (right). β-actin was included as an internal control. *P < 0.05, **P < 0.01, ***P < 0.001.

Processes involved in the EMT are closely correlated with cancer metastasis. We microscopically examined SHARP1-overexpressing EC cells to determine the effects of SHARP1 on cellular morphology. Ishikawa^SHARP1^ and HEC-1B^SHARP1^ cells were morphologically transformed toward epithelia compared with Ishikawa^Control^ and HEC-1B^Control^ cells (Figure 2A). This change was characterized by a loss of spindle-shaped morphology and a gain of cell-cell contacts, suggesting a phenotypic transition from mesenchymal to epithelial. To determine if this morphological transformation represented an EMT (as has been reported [27]), we analyzed levels of several proteins by western blotting. In Ishikawa^SHARP1^ and HEC-1B^SHARP1^ cells, levels of the epithelial marker E-cadherin were increased, whereas levels of the mesenchymal markers vimentin and N-cadherin were decreased, compared with controls (Figure 2B).

Because E-cadherin is transcriptionally repressed by the transcription factor snail [27], we determined the effect of SHARP1 on snail expression levels. Exogenous SHARP1 downregulated snail levels in Ishikawa^SHARP1^ and HEC-1B^SHARP1^ cells compared with controls (Figure 2B).

### SHARP1 overexpression suppresses the NOTCH1 pathway in EC cells

Activation of the Notch pathway plays a vital role in EMT during cancer progression by transcriptionally activating the gene snail [28,29]. We therefore assessed levels of NOTCH1, intracellular domain of NOTCH1 (ICN), its downstream genes HES1and its ligand jagged 1 in Ishikawa and HEC-1B cells. Interestingly, NOTCH1 levels were substantially downregulated by SHARP1 overexpression, as were ICN, HES1 and jagged 1 (Figure 2D). This further supported the hypothesis that SHARP1 inactivates Notch signaling and that the inactivation of Notch signaling might mediate the effect of SHARP1 on the EMT.

### Re-activation of NOTCH1 signaling induces migration and an EMT phenotype in SHARP1-overexpressing Ishikawa cells

**Figure 3:**
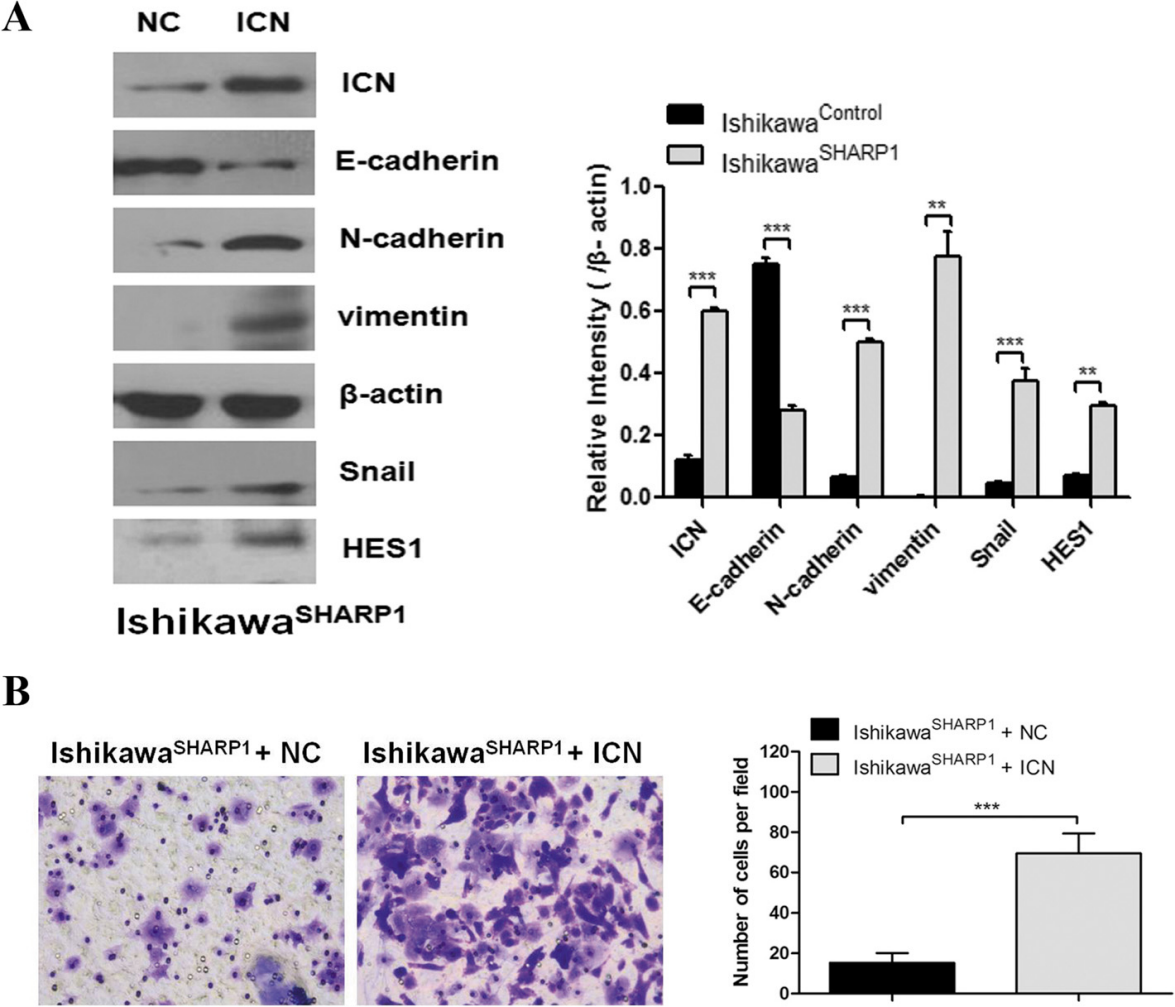
**Signaling through the NOTCH1 pathway induces migration and an EMT phenotype in *SHARP1 *-overexpressing Ishikawa cells. (A)** Western blot analysis of Ishikawa^SHARP1^ cells expressing the intracellular domain of NOTCH1 (ICN) or a negative control (NC). Protein levels of ICN, HES1 and EMT-related markers were analyzed by western blotting in Ishikawa^SHARP1^ cells (Left), and further quantified by densitometry of triplicate blots (right). β-actin was included as an internal control. **(B)** Trans-well migration assays involving Ishikawa^SHARP1^ cells expressing ICN or NC. Representative images were obtained at 200× magnification. Graph shows the average number of migrated cells (*n* = 5 images) for the two treatment groups. Data represent the mean ± SD from one representative experiment of three independent experiments. **P < 0.01, ***P < 0.001.

To determine whether SHARP1-mediated suppression of the EMT phenotype resulted from SHARP1’s ability to inhibit the NOTCH1 pathway, an intracellular domain of NOTCH1 (ICN) was expressed in Ishikawa^SHARP1^ cells via transient transfection, which exhibited a constitutively active function of the NOTCH1 receptor. ICN downregulated the epithelial marker E-cadherin, upregulated the mesenchymal markers vimentin and N-cadherin (Figure 3A), and increased the level of Snail and HES1. In addition, ICN promoted the migration of Ishikawa^SHARP1^ cells (Figure 3B).

### Verification of the SHARP1 effect on the Notch/EMT pathway in EC tissue specimens

A critical question that arose from our in vitro data was whether SHARP1 levels correlate with metastasis and expression of the EMT markers and Notch1 pathway genes in EC cells, as predicted by our hypothesis. To address this issue, we used a semiquantitative analysis of IHC staining to assess levels of these proteins in 20 specimens from 15 patients with EC. That stands for five specimens from each stage of EC (I–III) and five specimens of metastatic lymph nodes from the very same patients with stage-III EC.

**Figure 4:**
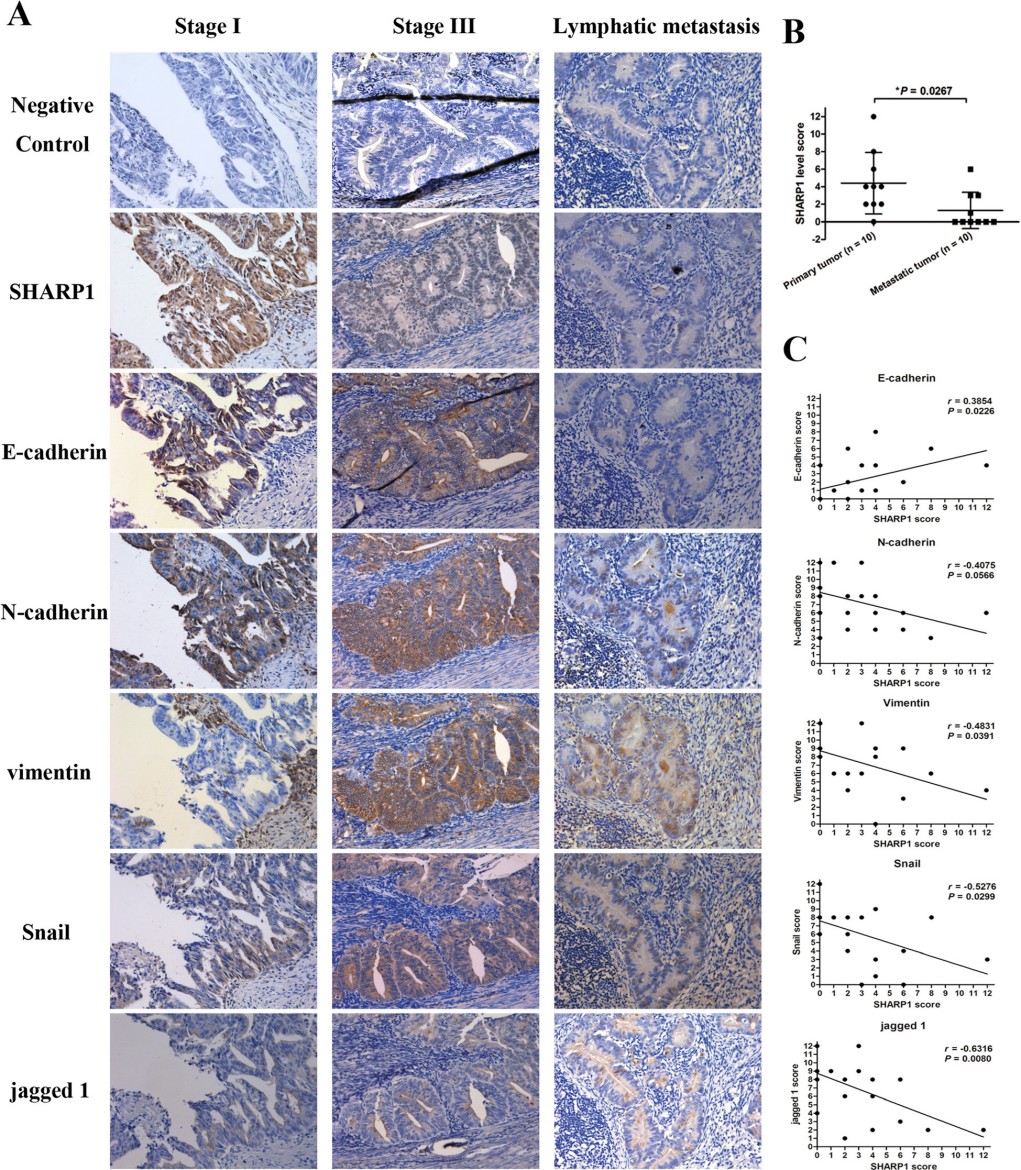
**Expression of SHARP1 and EMT markers in EC specimens. (A)** IHC analysis of SHARP1, E-cadherin, N-cadherin, vimentin, snail, and jagged 1 in EC (stage I or III) or lymphatic metastases, and IHC staining without primary antibody were used as negative control (magnification: 200×). **(B)** Levels of SHARP1 in primary and metastatic tumors determined by semiquantitative analysis of IHC staining (*P = 0.0267). **(C)** Expression correlations between SHARP1 and E-cadherin, N-cadherin, vimentin, snail, and jagged 1. Statistical analyses were performed using the Spearman’s correlation coefficient test.

IHC analysis confirmed that SHARP1 levels were significantly lower in metastatic EC tissues (*P* = 0.0267; Figure 4A and B). Moreover, SHARP1 levels positively correlated with E-cadherin levels (*P* = 0.0226) and inversely correlated with levels of vimentin (*P* = 0.0391), snail (*P* = 0.0299), and jagged 1 (*P* = 0.0080) (Figure 4A and C). And there was a trend of negative correlation between SHARP1 and N-cadherin levels (*P* = 0.0566).

## Discussion

Tumor invasion and metastasis are complex, multistep processes involving both genetic and epigenetic alterations. Consequently, cancer cells disseminate from the primary tumor and invade distant organs. Because tumor dissemination and metastasis are the leading causes of death in EC, elucidating the molecular mechanisms that underlie invasion and metastasis is important for developing new therapeutic strategies and improving clinical outcomes of patients with EC.

SHARP1 is a basic helix-loop-helix transcription factor that is involved in a number of cellular processes, including proliferation [9], apoptosis [30,31], differentiation [32], and circadian rhythms [33-35]. SHARP1 is generally believed to act as a tumor suppressor, and Montagner, et al. [11] recently showed that SHARP1 regulates the invasive and metastatic phenotype of triple-negative breast cancer cells by promoting degradation of hypoxia-inducible factors. In previous study, we showed SHARP1 suppresses angiogenesis of endometrial cancer by decreasing HIF-1α level [13]. In the current study, overexpression of SHARP1 suppressed EC-cell migration and invasion in vitro and tumor metastasis in vivo. Our data are consistent with previous findings and suggest for the first time that SHARP1 plays a critical role in tumorigenesis and acquisition of the metastatic phenotype in EC. However, the underlying mechanisms remain unknown and must be addressed by further investigation.

The EMT is a key event during cancer progression, leading to a more invasive, metastatic phenotype in human cancers, including EC [14,29]. At the molecular level, a variety of factors have been implicated in EMT. The loss of E-cadherin appears to be a crucial step, as this reduces cell-cell adhesion and destabilizes the epithelial architecture. Moreover, the protein snail, which is activated during the acquisition of EMT, plays a central role in repressing E-cadherin expression. Recent studies indicate that EMT status is associated with aggressive tumor characteristics and prognosis in EC [36,37]. However, the role of SHARP1 in this process remains unclear. Here we observed a morphological transformation in Ishikawa^SHARP1^ and HEC-1B^SHARP1^ cells with a concomitant increase in E-cadherin and reduction in N-cadherin and vimentin, suggesting a mesenchymal-to-epithelial transition in EC. Moreover, the reduced level of snail in these cells supports the idea that SHARP1 inhibits EMT in EC.

The Notch pathway is highly conserved and regulates cell fate specification, stem cell maintenance, and the initiation of differentiation in embryonic and postnatal tissues. Notch signaling also promotes EMT during cardiac development and tumor progression by inducing Snail, which subsequently downregulates cadherins [28]. Our data showed that the expression of NOTCH1 signaling pathway genes, Notch1, ICN, jagged 1 and HES1, was attenuated by SHARP1 overexpression in EC cells, indicating that SHARP1 inactivated the NOTCH1 pathway and suggesting that SHARP1-mediated suppression of NOTCH1 signaling contributes to suppression of EMT in EC. Future studies must determine whether SHARP1 regulates other signaling pathways capable of inducing EMT, such as the NFκB and WNT/β-catenin pathways.

Tumor-cell metastasis and invasion are responsible for most cancer-related mortalities. Invasive EC cells primarily metastasize to the lymphatic system, and we showed that SHARP1 overexpression decreased lymph-node metastases compared with primary tumors. Moreover, we detected a positive correlation between SHARP1 and E-cadherin levels and negative correlations between SHARP1 level and levels of vimentin, snail, and jagged 1. These results support our findings in EC cell lines. Although surgery is the standard treatment for patients with EC, patients with lymph-node or distant-organ metastases also require chemoradiotherapy. Thus, identifying biomarkers that define the metastatic potential of EC cells may help optimize treatment strategies. In this study, we found that evaluating SHARP1 levels in EC tissues (using IHC) effectively identified patients with EC who were at risk for lymph-node metastasis. Thus, SHARP1 may be a potential marker for EC metastasis and a target for therapeutic intervention.

## Conclusions

In summary, our results with EC cells show that SHARP1 suppressed migration, invasion, the EMT phenotype, and metastasis and that these effects involved downregulation of NOTCH1 signaling. Moreover, using EC tissue specimens we detected negative correlations between SHARP1 level and both lymphatic metastasis and markers of EMT. Our data suggest for the first time that impacts of SHARP1 on the NOTCH1/EMT system play a critical role in malignant progression and acquisition of metastatic phenotypes in EC. Thus, targeting SHARP1 could represent a new treatment option for preventing EC metastasis.

## Abbreviations

EC: Endometrial cancer; SHARP1: Enhancer-of-split and hairy-related protein 1; ICN: Intracellular domain of NOTCH1; EMT: Epithelial-mesenchymal transition; PBS: Phosphate-buffered solution; SCID: Severe combined immunodeficiency; HES1: Hes family basic helix-loop-helix transcription factor 1; qPCR: Quantitative real-time reverse transcription polymerase chain reaction assays; NS: Not significant.

## Competing interests

The authors have declared that no competing interests exist.

## Authors’ contributions

YL, XH, and HQ carried out the design of the experiments, performed most of experiments, and drafted the manuscript. ZC, WL and HW participated in the molecular biology experiments and statistical analysis. QC and FW participated in tumor pathological characterization. JW and MQ made the figures. XW was involved in financial support, the design of the experiments, data analysis, and final approval of the manuscript. All authors read and approved the final manuscript.

## Pre-publication history

The pre-publication history for this paper can be accessed here:

http://www.biomedcentral.com/1471-2407/14/487/prepub
